# Etymologia: *Anopheles culicifacies*

**DOI:** 10.3201/eid2808.211875

**Published:** 2022-08

**Authors:** Gaurav Kumar

**Affiliations:** National Institute of Malaria Research, Delhi, India

**Keywords:** Anopheles culicifacies, mosquitoes, Culex spp., malaria, vector-borne infections, wing morphology, George M.J. Giles, India

## *Anopheles culicifacies* [′ə′ nɒfɨliːz′ kyü-lə cifā-sh(ē-)ēz]

**Figure 1:**
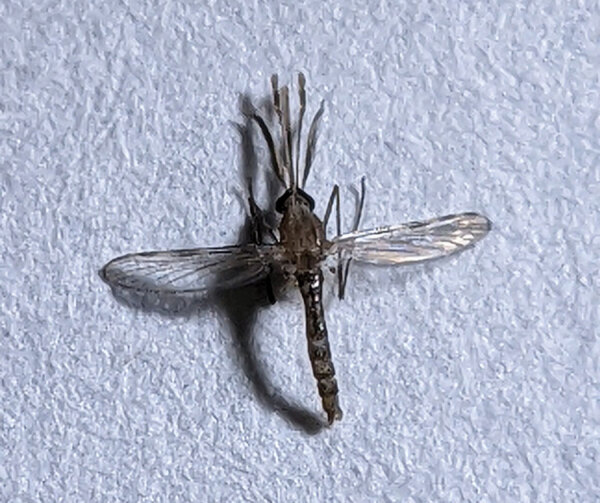
*Anopheles culicifacies* mosquito. Photograph courtesy Gaurav Kumar.

**Figure 2:**
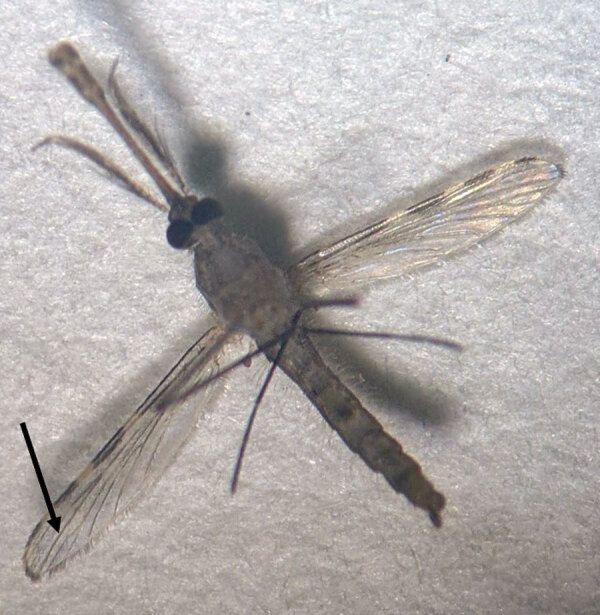
Wing morphology of *Anopheles culicifacies* mosquito showing the dark third vein (arrow). The length of the wing on the right is 2.5 mm. Photograph courtesy Gaurav Kumar.

In 1901, George Michael James Giles, a lieutenant-colonel and physician in the Indian Medical Service, described *Anopheles culicifacies*, which he collected from his guest house in Hoshangabad, India (Figure 1). This mosquito mimicked *Culex* spp. in facial appearance and resting posture (body angled to the surface they are resting on), prompting Giles to name it *Anopheles culicifacies* because of its culex (culici)‒like appearance (facies) (Figure 2).

*An. culicifacies* is the principle vector of malaria in India, contributing to >60% of malaria cases in this country annually. Therefore, ≈80% of the budget for malaria control in India is spent on control of this mosquito. Adults can be identified based on characteristic wing morphology (dark third vein) and palpi ornamentation (apical pale band is nearly equal to the pre-apical dark band).
